# Low dietary adherence after a positive food challenge in food allergic adults

**DOI:** 10.1002/clt2.12119

**Published:** 2022-02-18

**Authors:** Astrid Versluis, Thuy‐My Le, Francine C. van Erp, Mark A. Blankestijn, Geert F. Houben, André C. Knulst, Harmieke van Os‐ Medendorp

**Affiliations:** ^1^ Department of Dermatology/Allergology University Medical Centre Utrecht University Utrecht Utrecht The Netherlands; ^2^ TNO, Netherlands Organization for Applied Scientific Research Utrecht The Netherlands; ^3^ Saxion University of Applied Sciences, School of Health Enschede The Netherlands

**Keywords:** adults, dietary adherence, dietary advice, food allergy, food challenge

## Abstract

**Background:**

After a positive food challenge (FC), patients receive dietary advice regarding avoidance of the culprit food. We examined the frequency and variables associated with dietary adherence after a positive FC in adults.

**Methods:**

In this prospective daily practice study, adults with a positive FC were included. After every FC, dietary advice was given consisting of three options: (1) strict avoidance, (2) avoidance but products with precautionary allergen labelling (PAL) allowed and (3) (small) amounts allowed. Questionnaires about dietary adherence and associated variables were completed prior to and 6 months after the FC(s).

**Results:**

41 patients (with 58 positive FCs) were included. Overall, patients adhered to the advised diet after 31% of the FCs. After 33 FCs, the advice was strict avoidance, whereof 82% followed a less strict diet. After 16 FCs, the advice was avoidance but products with PAL allowed, whereof 19% followed a less strict and 25% a stricter diet. In 9 FCs with the least strict advice, “(small) amounts allowed’’, 67% followed a stricter diet. Three variables were associated with adherence: misremembering dietary advice, impaired health‐related quality of life (HRQL) on domain “Emotional impact’’ and the need for dietary change after the FC.

**Conclusion:**

After one third of the positive FCs, patients adhered to the dietary advice. Variables associated with adherence were misremembering dietary advice, impaired HRQL on domain “Emotional impact’’ and the need for dietary change after the FC. It seems important that healthcare professionals should more frequently apply adherence‐enhancing strategies to improve dietary adherence.

## 1 INTRODUCTION

Food allergy is an adverse immune response to food proteins that can cause symptoms involving skin, mucous membranes, gastro‐intestinal and respiratory tracts and the cardiovascular system.^1^ Diagnostics in patients with a suspected food allergy includes a detailed medical history, assessment of sensitization and a food challenge (FC). A double‐blind placebo‐controlled FC is the gold standard for diagnosing food allergy.^2^ After a positive FC, dietary avoidance of the culprit food is the key intervention.^1^ The dietary restrictions should be tailored to the individuals specific allergic and nutritional needs.^1^ For example, in patients with pollen‐food syndrome, which is common in adults, different fruits, nuts and vegetables may cause symptoms when eaten raw, but are tolerated when eaten cooked.^3^ It is necessary for each patient to receive counselling and education to manage the elimination of the culprit food(s) from their diet.^1^

Following the dietary advice is important to prevent accidental allergic reactions, unnecessary dietary restrictions, impairment of quality of life, costs and nutritional deficiencies.^1^, ^4^, ^5^, ^6^ Previous studies showed, remarkably, that food allergic children and adolescents often fail to adhere to dietary advice to avoid the culprit foods.^7^, ^8^, ^9^ In parents of children with a doctor‐diagnosed sea‐food allergy, it was shown that only one third adhered to the given dietary advice.^7^ In college students with self‐reported food allergies, only half of them always avoid the culprit food.^9^ And in adolescents (13–19 years of age) with a severe, doctor‐diagnosed food allergy, it was reported that 85% of them generally tried to avoid the food; however, less than half enquired about ingredients in restaurants (42%) or at friends' houses (35%). Only 16% of the adolescents were adherent to all aspects of self‐care investigated.^8^ Further, it has been shown that approximately half of adults with a doctor‐diagnosed food allergy experience on average two accidental allergic reactions per year, in some cases due to incorrect management of the advised dietary advice.^10^

Information about frequency and variables associated with adherence to dietary advice in adults with a doctor‐diagnosed food allergy is scarce. Therefore, this study investigated the frequency and variables associated with dietary adherence after a positive FC in adults.

## 2 METHODS

### 2.1 Study design, setting, study population and ethics

A daily practice study with a quantitative prospective design was carried out from 2014 till 2017 at the Department of Allergology/Dermatology of a tertiary referral center for food allergy in the Netherlands.

All patients (≥18 years) who underwent a positive FC with at least one of the 13 EU regulated allergenic foods (cereals containing gluten, crustaceans, eggs, fish, peanuts, soybeans, milk, nuts, celery, mustard, sesame seeds, lupin, molluscs) were included.

All patients gave written informed consent prior to inclusion. The Medical Ethics Review Committee of the University Medical Centre Utrecht confirmed on October 15, 2013 that the Medical Research Involving Human Patients Act (WMO) did not apply to the study (protocol number: 14‐237/C).

### 2.2 Standardized methods for food challenges and follow‐up care

Every patient underwent a standardized allergy work‐up. The first step included collection of a detailed medical and dietary history and assessment of sensitization (specific IgE and/or skin prick testing). Secondly, a FC was conducted, to confirm or rule out a food allergy, to assess severity of symptoms or to investigate thresholds.^1^ The food challenges were performed in an open or blinded manner and all ended with a daily normal dose of that food.^1^ Food challenges were conducted and interpreted by experienced staff, consisting of an allergy nurse, clinical nurse specialist, dietician and dermatologist in accordance with standardized procedures.^11^ Dietary advice was determined individually per patient by the experienced staff, based on sensitivity and severity of symptoms during the FC and each individual patient's history regarding intake of the challenged food in daily diet.^12^ There were three dietary advice options. Option 1: strict avoidance of the allergenic food and ingredients [including products with precautionary allergen labelling (PAL)]. Option 2: avoidance of the allergenic food and ingredients but products with PAL allowed. Option 3: (small) amounts of the allergenic food or ingredients allowed with dose adjustment based on complaints and on careful and complete evaluation [only in case of mild (mainly oral allergy) symptoms during FC and/or mild reaction to only a high dose].

**FIGURE 1:**
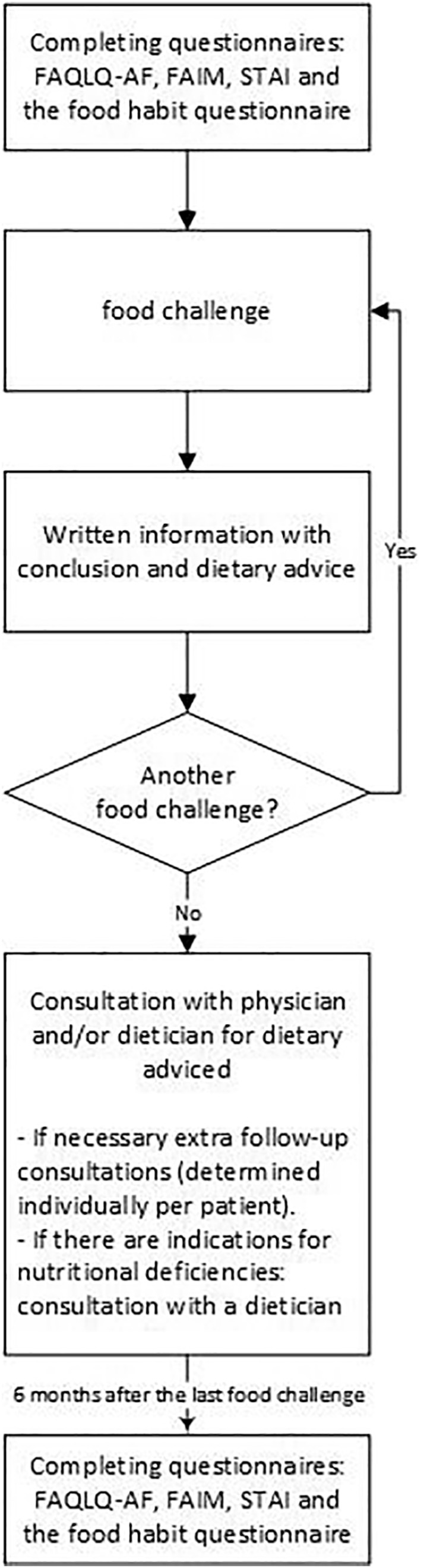
Flowchart of research procedure and standardized follow‐up care after a positive food challenge (FC)

After each positive FC, patients received a standardized follow‐up consisting of written information about the conclusion and dietary advice, and a consultation with the physician and/or dietician when all tests had been performed. If indicated, additional follow‐up consultations could be scheduled (Figure 1).

### 2.3 Outcome measures

The primary outcome measure was frequency of dietary adherence. Dietary adherence was defined as ‘consequently following dietary advice’.

The secondary outcome measure was the association of a number of variables with dietary adherence, including: consultation with a dietician instead of a physician during follow‐up, accurate recollection of the prescribed dietary advice at follow‐up, the need for a dietary change after the FC (if the habitual diet prior to the FC differed from the advised diet after the FC), if the type of food challenged was nuts/peanuts versus other foods, if the patient experienced accidental allergic reactions during follow‐up, the method of FC (single/double blind vs. open), age (adolescent vs. adult), the number of positive food challenges (one vs. more than one), health‐related quality of life (HRQL) at baseline and state and trait anxiety at baseline. Furthermore, reasons for non‐adherence were studied in patients who consciously failed to adhere to the advised diet.

### 2.4 Data collection

Patients were asked to complete four questionnaires prior to and 6 months after the last FC, consisting of: the food habit questionnaire, the Food Allergy Quality of Life Questionnaire‐Adult Form (FAQLQ‐AF),^13^ the Food Allergy Independent Measure (FAIM)^14^ and the State‐Trait Anxiety Inventory (STAI)^15^ (Figure 1). The food habit questionnaire included items about avoidance of the challenged food(s). This questionnaire was developed by a multidisciplinary team consisting of an allergist, dietician, nurse scientist and clinical nurse specialist. Feasibility of the questionnaire was achieved by conducting a pilot in small group of patients who underwent a FC at the day care unit. The questionnaire filled in 6 months after the last FC included additional items about what dietary advice patients thought they had received after the FC, whether patients experienced accidental food allergic reactions during the follow‐up period and patients' reasons in the event that they consciously chose not to adhere to the received dietary advice. The FAQLQ‐AF consisted of four domains (Risk of accidental exposure, Emotional impact, Allergen avoidance‐dietary restrictions and Food allergy‐related health) comprising a total of 29 items about food allergy specific quality of life. The total score ranged from 1 (no impairment) to 7 (maximal impairment).^13^ The FAIM consisted of 4 items about patients' perceived food allergy severity and food allergy related risks. The total score varies from 1 (limited severity perception) to 7 (greatest severity perception).^14^ The STAI consisted of 40 items and covered aspects of state anxiety (in the specific situation of eating the food the patient is allergic to) and trait anxiety (feelings of stress, worry, discomfort, etc. that a person experiences on a daily basis). The score varies from 20 (minimal anxiety) to 80 (maximal anxiety) in both state and trait anxiety.^15^ The Dutch validated versions of the FAQLQ‐AF, FAIM and STAI, were used and the scores were calculated using standardized methods.^13^, ^14^, ^15^

Additionally, patients completed a questionnaire about atopic comorbidities and educational level. Other characteristics of patients and food challenges were collected from the patients' medical records. The severity of allergic reactions was classified based on type of symptoms: local oral symptoms were classified as “mild”, symptoms from skin and mucous membranes and/or gastro‐intestinal tract as “moderate” and respiratory and/or cardiovascular symptoms as “severe”.

### 2.5 Sample size and statistical methods

We did not carry out a sample size calculation, but all patients undergoing one or more positive food challenges over a period of 3 years and who met the inclusion criteria were asked to participate in the study.

Outcome data regarding frequency, variables associated with dietary adherence and reasons for non‐adherence, were analysed using descriptive statistics. Depending on level of measurement, frequency (n/%) or mean (SD) were used.

Differences between patients who adhered to the dietary advice, followed a stricter diet than advised or followed a less strict diet than advised with regard to variables associated with dietary adherence were analysed using the Fisher‐Freeman Halton test or Kruskal–Wallis test depending on level of measurement and data distribution. Some variables were analyzed per patient (instead of per FC). In these factors, group classification (follows diet as advised, follows a stricter diet than advised and follows a less strict diet than advised) was based on dietary adherence after the most severe (and in case of similar severity, the first) FC of the patient.

A *p*‐value <0.05 was considered statistically significant. Data were analyzed using IBM SPSS Statistics 25 (IBM Corporation).

## 3 RESULTS

### 3.1 Characteristics of patients and food challenges

In this study, a total of 41 patients were included, who underwent a total of 58 food challenges with a positive outcome, confirming the food allergy. The majority of patients were female (71%) and the mean age was 33 years (SD: ±12, min‐max: 19–61). Most patients had atopic comorbidity: asthma (68%), atopic dermatitis (58%, 23/40, *n* = 1 missing) and/or allergic rhino conjunctivitis (88%). The majority of the patients underwent one positive FC (71%), and the other patients underwent 2 (17%) to 3‐4 (12%) positive food challenges. The mean time between FC and evaluation of dietary adherence was seven months (SD: ±3, min‐max: 5–16, missing: *n* = 3).

Of the total 58 positive food challenges, most commonly challenged foods were nuts (54%) and peanut (17%). The allergic reactions during the food challenges were mainly moderate (48%) or severe (35%; Table 1).

**TABLE 1:** Characteristics of food challenges

	All food challenges *n* =v58
	*n* (%)
Food challenged:	
‐ Nuts^a^	31 (54)
‐ Peanut	10 (17)
‐ Hen’s egg	5 (9)
‐ Sesame	4 (7)
‐ Cow’s milk	4 (7)
‐ Other^b^	4 (7)
The method of the food challenge:	
‐ Single/double blind	42 (72)
‐ Open	16 (28)
Severity of reaction during food challenge^c^:	
‐ Mild	10 (17)
‐ Moderate	28 (48)
‐ Severe	20 (35)

^a^ Nuts includes: walnut (*n* = 12), hazelnut (*n* = 11), cashew nut (*n* = 5), almond (*n* = 3).

^b^ Other includes: shrimp (*n* = 1), grains (*n* = 2) and soy (*n* = 1).

^c^ Mild: local oral symptoms, moderate: symptoms from skin and mucous membranes and/or gastro‐intestinal tract and severe: respiratory and/or cardiovascular symptoms.

After almost two thirds of the food challenges (66%), patients received dietary advise via standardized follow‐up care (via written information and consultation with a physician and/or dietician) and in the other food challenges, only via consultation with a physician and/or dietician (17%) or only via written information (17%).

### 3.2 Only one third of the patients adhered to the dietary advice

After the positive food challenges, patients received dietary advice, consisting of the three options: (1) strict avoidance of the culprit food, (2) avoidance but products with PAL allowed and (3) (small) amounts allowed. Patients adhered to the advised diet after 31% (18/58, 95% CI: 20%–45%) of all food challenges.

**FIGURE 2:**
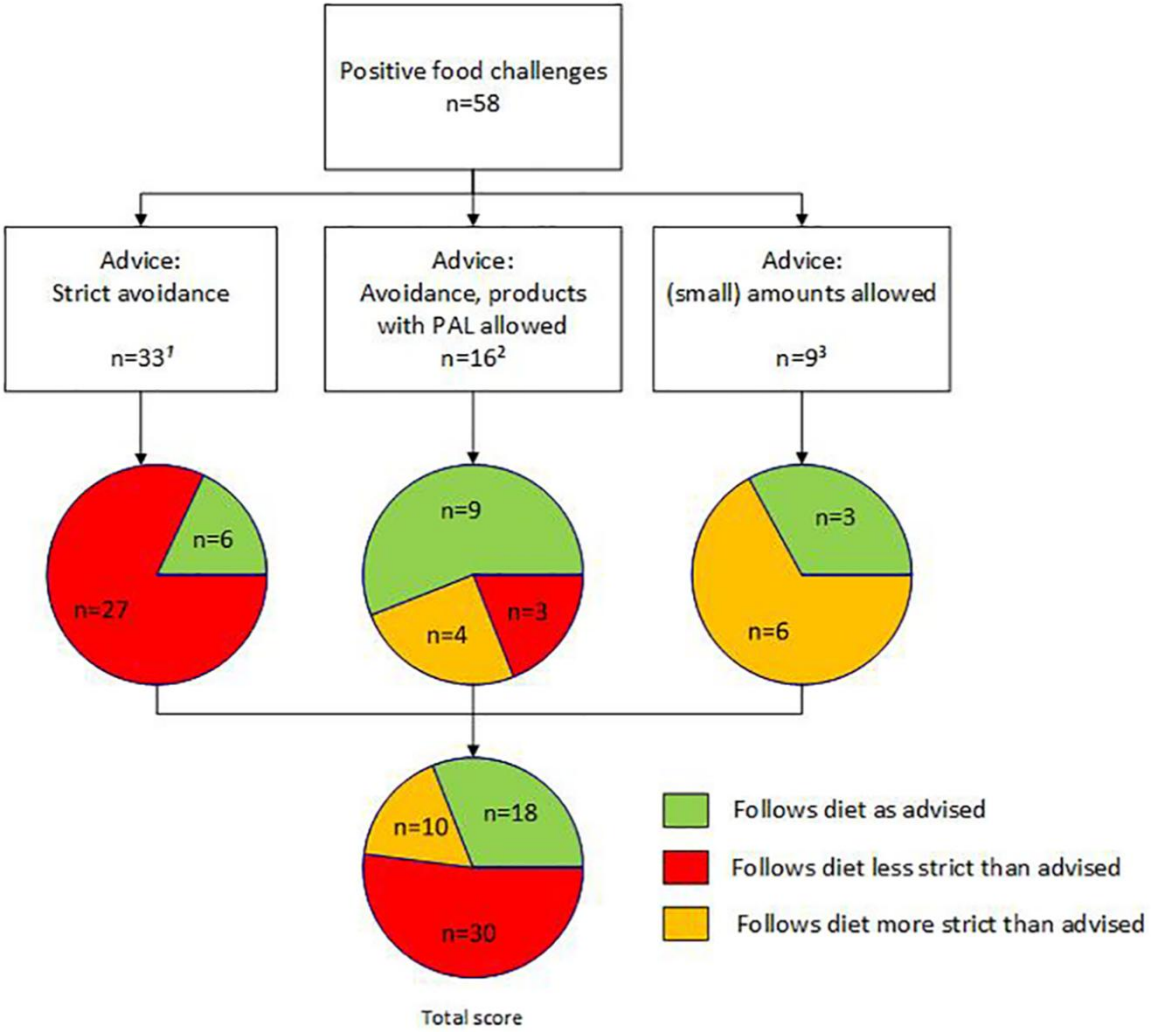
Dietary adherence. ^1^Types of food allergen: peanut: n = 7, hazelnut: n = 3, nuts (excl. hazelnut): n = 12, cow's milk: n = 3, hen's egg: n = 5, sesame: n = 3. ^2^Types of food allergen: peanut: n = 3, hazelnut: n = 4, nuts (excl. hazelnut): n = 6, cow's milk: n = 1, shrimp: n = 1, grain: n = 1. ^3^Types of food allergen: hazelnut: n = 4, nuts (excl. hazelnut): n = 2, soy: n = 1, sesame: n = 1, grain: n = 1

After 33 food challenges, the dietary advice was strict avoidance of the allergenic food and ingredients. In the vast majority of this group (82%, 27/33), a less strict diet was followed (Figure 2). After 16 food challenges, advice to follow a less strict diet was given, namely to avoid the allergenic food and ingredients, but not products with PAL. In almost half of these cases (44%, 7/16), the dietary advice was not followed: in 19% (3/16) a less strict diet was followed and 21% (4/16) a stricter diet.

In nine food challenges with mild allergic reactions, the dietary advice was that (small) amounts of the allergenic food or ingredients were allowed because of the mildness (mainly oral allergy) of the symptoms during FC and/or mild reaction only in the event of a high dose. In this group, after two‐thirds (6/9) of the food challenges a stricter diet than advised was followed.

### 3.3 Variables associated with adherence to dietary advice

We examined which variables were associated with dietary adherence. Table 2 shows the association between different variables and adherence to dietary advice, comparing the patient groups who: (a) followed diet as advised, (b) followed a stricter diet and (c) followed a less strict diet. Comparing these three groups gives insight as to whether these variables are associated with dietary adherence and whether it might lead to a less or more strict diet.

**TABLE 2:** Variables associated with adherence to dietary advice

Variables	Follows diet as advised	Follows diet stricter than advised	Follows diet less strict than advised	Comparison of group: adherence, stricter diet and less strict diet
	Per food challenge			
	*N* (%)	*N* (%)	*N* (%)	*p*‐value^a^
Was the prescribed dietary advice accurately recollected at follow‐up (*n* = 56):				0.01
Yes	12 (67)	3 (33)	25 (86)	
No	6 (33)	6 (67)	4 (14)	
Was a dietary change was needed after the FC (*n* = 33)				0.08
Yes	5 (46)	7 (88)	12 (86)	
No	6 (54)	1 (13)	2 (14)	
Follow‐ up consultation with (*n* = 48)^b^:				1.00
Dietician	11 (73)	6 (75)	19 (76)	
Physician	4 (27)	2 (25)	6 (24)	
Type of food challenged (*n* = 58):				0.59
Peanut or nuts	11 (61)	8 (80)	22 (73)	
Other foods	7 (39)	2 (20)	8 (27)	
Method of the food challenge (*n* = 58):				0.45
Single/double blind	12 (67)	9 (90)	21 (70)	
Open	6 (33)	1 (10)	9 (30)	

	Per patient, adherence after FC with most severe outcome			
	*N* (%)	*N* (%)	*N* (%)	*p*‐value^a^
Did a food‐induced allergic reaction(s) occur during follow‐up (*n* = 41):				0.36
Yes	5 (33)	5 (63)	10 (56)	
No	10 (67)	3 (38)	8 (44)	
Age (*n* = 40)^c^				1.00
Adolescent (≤24 years of age)	4 (27)	2 (25)	5 (29)	
Adults	11 (73)	6 (75)	12 (71)	
Number of positive food challenges (*n* = 41)				0.61
1	10 (67)	7 (88)	6 (33)	
>1	5 (33)	1 (13)	12 (67)	

HRQL and anxiety before food challenge(s)	Mean (SD)	Mean (SD)	Mean (SD)	*p*‐value^g^
	*N* = 14‐15^d^	*N* = 6‐7^e^	*N* = 16‐18^f^	
Food allergy related quality				
Total score	4.0 (1.4)	3.7 (1.2)	4.4 (1.3)	0.30
Domain Risk of accidental exposure	4.2 (1.6)	3.6 (1.7)	4.6 (1.3)	0.44
Domain Emotional impact	4.1 (1.5)	3.5 (1.2)	4.9 (1.3)	0.02
Domain Allergen avoidance‐dietary restrictions	3.8 (1.5)	4.0 (1.3)	4.0 (1.6)	0.84
Domain Food allergy‐related health	4.3 (1.8)	3.6 (1.4)	4.1 (1.7)	0.62
FAIM	3.4 (1.0)	3.0 (0.6)	3.6 (1.0)	0.22
STAI: State anxiety	35.1 (12.1)	33.0 (12.1)	30.6 (9.7)	0.69
STAI: Trait anxiety	35.0 (8.3)	29.2 (9.3)	36.2 (9.0)	0.17

^a^ Statistical test used: Fisher‐Freeman‐Halton Test.

^b^ Patient who received dietary advice via consultation.

^c^ Missing: *n* = 1.

^d^ Missing: *n* =  0‐1.

^e^ Missing: *n* = 1‐2.

^f^ Missing: *n* = 0‐2.

^g^ Statistical test used: Kruskal Wallis test.

The first variable investigated was accurate recollection of the prescribed dietary advice. Figure 2 shows the prescribed dietary advice. In the follow‐up questionnaire, patients self‐reported the dietary advice they received per FC. Almost one third of all patients (29%, 16/56, missing *n* = 2) misremembered the prescribed dietary advice. Patients who followed a stricter diet most often misremembered the diet (67%), compared to patients who adhered to the diet (33%) and patients that followed a less strict diet (14%; *p* = 0.01).

Secondly, the variable “the need for a dietary change after the FC” was investigated. In more than two thirds of the food challenges (72%, 24/33, missing *n* = 15) the advised diet after the FC differed from the habitual diet prior to the FC. In patients following a stricter diet and also in patients who followed a less strict diet, the advised diet after the FC more often differed from the habitual diet prior to the FC, compared to patients who adhered to the advised diet (88% and 86% vs. 46%, *p* = 0.08).

Further, the variable HRQL and anxiety at baseline was investigated, measured with the FAQLQ‐AF, FAIM and STAI. In patients following a less strict diet, the baseline score of FAQLQ‐AF domain Emotional impact was more impaired compared to patients who adhered to the advised diet or followed a stricter diet (*p* = 0.02). No differences between the three patient groups was found in the other FAQLQ‐AF domains, FAIM and STAI (Table 2).

No difference was found between the three patient groups with regard to the healthcare professional that gave dietary advice (dietician vs. physician, *p* = 1.00), occurrence of accidental food‐induced allergic reactions during follow‐up (*p* = 0.36), the type of food challenged (peanuts/nuts vs. other foods; *p* = 0.59), the method of FC (single/double blind vs. open, *p* = 0.45), age (adolescent vs. adult, *p* = 1.00) and the number of positive food challenges (one vs. more than one, *p* = 0.61).

### 3.4 Non‐adherence was a conscious choice in more than one third of the patients

Of the patients who did not adhere to the dietary advice, more than one third (35%, 13/37, *n* = 3 missing) reported that this was a conscious choice. The other patients (65%, 24/37) did not mention such a conscious choice for non‐adherence.

Most of the patients who made this conscious choice, received advice to strictly avoid the food but followed a less strict diet (77%, 10/13) with two different reasons: a strict diet led to too many restrictions in diet (4/10) and using products with PAL was expected to be safe (4/10). In two cases, no reason was recorded. The other three patients (23%), received the advice that (small) amounts were allowed but they consciously chose to avoid the food, because they expected allergic complaints upon consuming the food.

## 4 DISCUSSION

In this study, we showed that in only one third of the positive food challenges, patients adhered to the dietary advice. Variables associated with adherence were: misremembering dietary advice, an impaired HRQL on domain Emotional impact and the need for a dietary change after the FC.

It is remarkable that dietary adherence after a positive FC in adults is low, despite all patients having been given dietary advice. Two previous studies investigating dietary adherence in children and adolescents with a doctor‐diagnosed food allergy showed that only one third of the parents of children with a sea‐food allergy adhered to the dietary advice and that less than half of the adolescents enquired about ingredients in restaurants or when visiting the house of a friend.^7^, ^8^ To our knowledge, this is the first study to show low dietary adherence in food allergic adults. The low frequency of dietary adherence is a major concern because of the risk of accidental allergic reactions in case of a less strict diet and the risk of unnecessary product avoidance and social impairment in case of a stricter diet than advised.^5^, ^6^, ^10^ Non‐adherence is also a well‐recognized problem in other types of medical advice; for example, in adherence to medication and in following dietary and lifestyle changes in other diseases.^16^, ^17^, ^18^

In our study, dietary adherence was lowest in patients who received advice to strictly avoid a food. Strict avoidance meant that the culprit food including products with PAL should be avoided. Several factors might negatively influence the adherence to the advice to avoid these products. First, patients are confronted with unstandardized presentation of information on food labelling, which is often unclear, with low readability and clarity and consequently difficulty in interpretation.^19^, ^20^ Second, PAL is increasingly present on products, strongly restricting food choices.^4^ Third, some patients estimate the risk, based on product name and brand and prior experiences.^21^ Finally, even for products without PAL, there is no guarantee that these are without allergens, adding to the confusion.^22^ Overall, patients who have to avoid products with PAL face many obstacles, so healthcare professionals should guide and support patients to better‐deal with these difficulties. Regulations of food labelling and PAL would help food allergic patients to better manage their diet.

In general, food allergic patients are advised to strictly avoid the culprit food.^23^ However, it is not necessary for all food allergic patients to completely avoid the culprit food. Sicherer et al.^12^ reported in a review, that, in patients who are not highly allergenic, options such as usage of products with PAL or allowing a small amount of the culprit food may be considered individually per patient. In our study one of the following options for dietary advice was given after the FC: (1) strict avoidance (33/58), (2) avoidance but products with PAL allowed (16/58) and (3) (small) amounts allowed (9/58). Option 2 is mainly advised to patient with mild/moderate complaints who already use products with PAL for a longer period, without complaints. Currently, the Ad Hoc Joint The Food and Agriculture Organization of the United Nations/Worlds Health Organization Expert Consultation on Risk Assessment of food allergens works on a more accurate way of precautionary food labelling,^24^ which is already implemented by some food producers. Due to this developments, it seems more and more needed to advice a strict diet in patients who previously received the advice to avoid the food but who were allowed to use products with PAL.

We identified three variables that are associated with dietary adherence. The first was ‘‘misremembering the advised diet’’. In our study 29% misremembered the prescribed dietary advice. A previous study in children with a sea‐food allergy showed that almost one‐quarter of the parents were unable to correctly recall the dietary advice.^7^ Poor and inaccurate patient recollection of medical information is a well‐known problem.^25^, ^26^ The second variable was the need for a dietary change after the FC. Our results indicate that this is a factor in both patients who follow a stricter and a less strict diet as advised. It is known that changing dietary behavior is challenging.^27^ Conducting a qualitative study in which patients are interviewed about this topic seems valuable to generate more insight in this variable. The third variable we found was that the HRQL domain Emotional impact was more impaired in patients who followed a less strict diet than advised. However, most patients who followed a less strict diet had a severe food allergy. So, HRQL might be indirectly associated with dietary adherence via having a severe food allergy, which itself is shown to negatively impact HRQL.^28^ Furthermore, it is reported that food challenges are associated with improvement of HRQL.^29^ Therefore, future research on this topic with repeated measures of HRQL seems valuable to get more insight into the relation between HRQL and dietary adherence. The sample‐size of our study was too small to further analyze the relationship between adherence, HRQL and severity using a multivariate model. Remarkably, no association was found between accidental allergic reactions and dietary adherence. However, we do not know if patients adapted their diet after experiencing a reaction, which would bias this result. In addition, literature showed that accidental allergic reaction often occur after not following the advised diet.^10^, ^20^ In summary, several variables might be associated with dietary adherence. It seems important that healthcare professionals consider these variables when giving advice and guidance about dietary restrictions. Future research should give more insight into additional variables that could be associated with dietary adherence, for example, methods used for diagnostics, the indication for the FC, severity of (accidental) reactions and the type of food allergen. Moreover, future research on the occurrence of accidental food‐induced allergic reactions during follow‐up seems needed, excluding the possible bias of patients adapting their diet after experiencing a reaction.

Our results indicate that patients who receive standardized follow‐up care after a positive FC(s), still frequently fail to adhere to dietary advice. This is disappointing and it indicates that the given follow‐up care is not sufficient. The follow‐up care given in our study was largely consistent with the international food allergy guideline of Muraro et al.^1^ which reports that education about risky situations, reading labels, the regulation of precautionary labels and possible substitute food products is essential for an effective long‐term elimination diet in food allergic patients. Different intervention strategies could be useful. It has been shown that parents of food allergic children benefit from food allergy management curriculums, with preferably a variety of educational materials.^30^, ^31^ An online self‐management program for food allergic patients can be used in addition to face‐to‐face consultations.^32^ Combined interventions seem to be most beneficial in achieving adherence. For example, education, supporting, building a trusting relationship, personalized care, shared decision‐making, evaluation and use of different tools (e.g. mobile apps, video, written materials).^33^, ^34^, ^35^, ^36^, ^37^ With regard to dietary advice after a positive FC, more frequent follow‐up consultations mainly focusing on imparting knowledge, supporting patients to adhere to their diet and discussing obstacles and barriers seem important, preferably always with the same healthcare professional.^1^, ^35^, ^36^ More insight about intervention strategies which are effective in enhancing dietary advice in food allergic adults is needed.

A limitation of this study was that it was conducted in a tertiary center with patients with a history of more severe food allergic reactions. This could have the effect of restricting the generalizability of our data to the general food allergic population. Furthermore, one third of the patients did not receive dietary advice via the standardized follow‐up care (i.e. 17% only via written information and 17% only via consultation with a physician and/or dietician). However, when comparing patients who had received standardized follow up care versus those who had not with regard to dietary adherence, no differences were found. Furthermore, our definition of dietary adherence was strict. If we had defined dietary adherence as ‘not following dietary advice one or less times per month’, dietary adherence would have been slightly higher: in patients with a strict diet 21% instead of 18% and in patients with dietary advice to avoid the food but products with PAL allowed 75% instead of 56%. A study about dietary adherence in parents of sea‐food allergic children also used the stricter definition that dietary advice should be followed all the time.^7^ Furthermore, the small sample size limits the power of the subgroup analysis and the generalizability of the results. A strength of this study was the prospective study design and use of validated questionnaires (with the exception of the food habit questionnaire), which contributed to the reliability of our results. An additional advantage of this study was that diagnosis and dietary advice was based on a FC. If only one third of the patients that experienced the severity of the reaction during a FC adhered to the dietary advice, it is the question whether dietary adherence is even worse in patients that are only diagnosed by history and sensitization. It would be interesting to investigate this in future studies.

In conclusion, patients adhered to the dietary advice after only one third of the positive food challenges. Variables associated with adherence were misremembering dietary advice, an impaired HRQL on domain Emotional impact and the need for a dietary change after the FC. Our results indicate that it is important for healthcare professionals to more frequently apply adherence‐enhancing strategies in order to improve dietary adherence.

## CONFLICT OF INTEREST

All authors declare that they have no conflicts of interest.

## AUTHOR CONTRIBUTIONS

**Astrid Versluis:** Conceptualization; Data curation; Formal analysis; Investigation; Methodology; Project administration; Resources; Writing – original draft. **Thuy‐My Le:** Supervision; Writing – review & editing. **Francine C. van** **Erp:** Conceptualization; Methodology; Writing – review & editing. **Mark A. Blankestijn:** Data curation; Writing – review & editing. **Geert F. Houben:** Supervision; Writing – review & editing. **André C. Knulst:** Conceptualization; Investigation; Methodology; Supervision; Writing – review & editing. **Harmieke van** **Os‐ Medendorp:** Conceptualization; Formal analysis; Methodology; Supervision; Validation; Writing – review & editing.
